# Interfacial Engineering of Semiconductor–Superconductor Junctions for High Performance Micro-Coolers

**DOI:** 10.1038/srep17398

**Published:** 2015-12-01

**Authors:** D. Gunnarsson, J. S. Richardson-Bullock, M. J. Prest, H. Q. Nguyen, A. V. Timofeev, V. A. Shah, T. E. Whall, E. H. C. Parker, D. R. Leadley, M. Myronov, M. Prunnila

**Affiliations:** 1 VTT Technical Research Centre of Finland, P.O. Box 1000, FI-02044 VTT Espoo, Finland; 2 Department of Physics, University of Warwick, Coventry CV4 7AL, UK; 3 Low Temperature Laboratory (OVLL), Aalto University School of Science, PO Box 13500, FI-00076 Aalto, Finland

## Abstract

The control of electronic and thermal transport through material interfaces is crucial for numerous micro and nanoelectronics applications and quantum devices. Here we report on the engineering of the electro-thermal properties of semiconductor-superconductor (Sm-S) electronic cooler junctions by a nanoscale insulating tunnel barrier introduced between the Sm and S electrodes. Unexpectedly, such an interface barrier does not increase the junction resistance but strongly reduces the detrimental sub-gap leakage current. These features are key to achieving high cooling power tunnel junction refrigerators, and we demonstrate unparalleled performance in silicon-based Sm-S electron cooler devices with orders of magnitudes improvement in the cooling power in comparison to previous works. By adapting the junctions in strain-engineered silicon coolers we also demonstrate efficient electron temperature reduction from 300 mK to below 100 mK. Investigations on junctions with different interface quality indicate that the previously unexplained sub-gap leakage current is strongly influenced by the Sm-S interface states. These states often dictate the junction electrical resistance through the well-known Fermi level pinning effect and, therefore, superconductivity could be generally used to probe and optimize metal-semiconductor contact behaviour.

The quality of the electrical contact between a semiconductor and a metal electrode is one of the key process elements in building high performance microelectronic circuits^1^,^2^. For transistors, the specific contact resistance needs to be sufficiently low to maximize drive currents and extensive efforts have been devoted to this topic since the dawn of semiconductor physics and integrated circuits. New materials (like 2D materials (graphene) and nanotubes) that are contenders to replace canonical semiconductors bring in new challenges to this field^3^,^4^,^5^. For example, one of the major obstacles, before graphene electronics becomes truly a viable high speed technology, is how to produce reliable low resistance contacts between 3D metal electrodes and 2D graphene.

Metal-semiconductor junctions can also have an active function, the classic example being the Schottky diode, which relies on the rectifying properties of the metal-semiconductor junction in the thermionic emission limit. Involving a similar physical process they can also be used as electro-thermal elements which allow a cooling heat flux due to electron energy filtering. One example is a Schottky junction in the tunnelling limit with temperature below the superconducting critical temperature of the metal electrode (see Fig. 1a,b). Such a semiconductor-superconductor (Sm-S) cooler junction introduces strong energy filtering for the tunnelling electrons due to the superconducting gap and the sharp peaks in the quasiparticle density-of-states (DOS) around the gap (see Fig. 1b). The Sm-S cooler junction^6^ is the counterpart of the fully metallic device based on normal metal-insulator-superconductor (NIS) tunnel junctions^7^,^8^. Sm-S and NIS junctions provide an effective laboratory to study non-linear electro-thermal effects, and they are envisioned to be utilized in high sensitivity bolometer devices and electronic cooler platforms^9^,^10^,^11^,^12^. The silicon-based Sm-S cooler junctions, with the Schottky tunnel barrier replacing the insulator tunnel barrier, were originally introduced to improve certain features of NIS based devices^6^. It was anticipated that the Schottky barrier could be free from the unwanted leakage and pinhole effects that were present in large scale, high transparency NIS junctions. Another advantage over the NIS devices, is that the unwanted parasitic phonons-to-electrons heat back-flow in Sm-S cooler devices is significantly smaller than in NIS coolers, due to the weaker electron-phonon coupling in semiconductors^13^,^14^,^15^. Coupled with the advanced processing infrastructure of Si-based devices the Sm-S junctions were anticipated to take superconductive junction coolers to a whole new technological level, with the capability to build large scale integrated cooler platforms for low temperature sensors and devices.

However, it turned out that the high transparency (low resistance) Sm-S junctions that are needed for efficient coolers, did not behave according to the expectations^16^,^17^. They suffered from significant sub-gap leakage, which can be described phenomenologically by smearing of the ideally sharp density of states in the superconductor (see Fig. 1c). Due to this, the field of Sm-S coolers did not flourish and the problems with leakage remained neither understood nor solved. In this work, we successfully tackle both of these items. We demonstrate the first high transparency and low leakage Sm-S junctions and provide an explanation of the physics behind the sub-gap leakage effect in Sm-S junctions. A low leakage junction is achieved by introducing an additional insulator tunnel barrier between the S (Al) and Sm (n+ Si) electrodes and, thereby, creating a superconductor-insulator-semiconductor (SISm) cooler junction. Despite the introduction of the insulating barrier (SiO_2_) the junction resistance remains low, which is attributed to Fermi level de-pinning and dopant segregation effects. Our results indicate that the sub-gap leakage is due to dopants in the tunnel Schottky barrier and, especially, due to surface states present at the Si-Al interface (Fig. 1c,d). The added SiO_2_ barrier effectively passivates the junction, removing both leakage channels (Fig. 1e,f). The main outcome of this work is the demonstration of low leakage high cooling power Sm-S devices, which can be utilized in large scale microcooler platforms and bolometers. Sm-S hybrids are also important for the emerging field of Majorana fermion quantum circuits^18^. In broader scope, our results are strongly linked to the physics of semiconductor-metal junctions and one of the key observations is that a metal electrode in the superconducting state acts as a sensitive probe to the metal-semiconductor surface states, which often dictate the junction resistance through the Fermi level pinning effect.

## Results

### Tunnel junction devices

To improve the Sm-S junction quality, we have investigated different junction processing methods, with and without *in-situ* oxidation. The processes have been used for two different types of Sm-S cooler devices: unstrained with implanted wells in bulk Si and strained silicon (sSi) with degenerately doped epi-layer. The unstrained bulk Si cooler samples, Si-1 and Si-2, consist of degenerately doped Si:P wells in a Si substrate, which define the active Si island, see Fig. 2a for sample layout. The junctions were defined by opening contact vias through an isolating oxide layer, see Fig. 2b. The sSi cooler consists of a 30 nm thick strained Si layer grown on a relaxed Si_1-x_Ge_x_ alloy buffer layer. A biaxial tensile strain is induced via the lattice mismatch between the silicon and the SiGe alloy. The degenerately doped layer is etched to form an isolated mesa structure and the strained Si island is contacted with Al electrodes to form the junction, see Fig. 2c,d.

For all samples, the Si was treated using Hydrofluoric (HF) acid to remove the native oxide and hydrogenate the Si surface. Sample Si-1 is used as the unstrained control, with Al deposition after the HF treatment to form Sm-S junctions. The unstrained sample Si-2 and the strained sample sSi were annealed and oxidized at 550 ^o^C *in-situ* in the sputtering system, before the Al deposition, which is intended to create a controlled oxide barrier in the interface, creating semiconductor-insulator-superconductor (SmIS) junctions. As a control for the sSi cooler we refer to earlier work^19^, where device geometry was the same, but the junctions were not subjected to our *in-situ* oxidation technique. In order to generate a sample with a high interface state density, we prepared one set of devices by damaging the Si surface with Ar plasma before the Al deposition. Fabrication details and sample parameters can be found from the Supplementary Material. The most relevant junction parameters together with earlier literature are listed in Table 1.

### Electronic properties

The current *I* through the superconducting tunnel junction is commonly described by the relation^9^,^10^


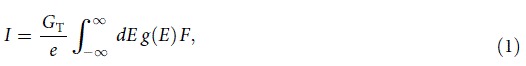


where *G*_T_ = (*R*_c_/*A)*^*−1*^ is the tunnel conductance, *e* is the elementary charge, *E* is the energy and *g(E)* is the superconducting density-of-states (DOS). Here *R*_*c*_ is the characteristic junction resistance and *A* is the area of the junction. The function *F* is the combined Fermi-Dirac distribution of the superconducting and semiconducting materials


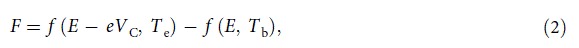


where *V*_c_ is the voltage over the junction, *T*_e_ is the electron temperature of the semiconductor, *T*_b_ is the bath temperature and *f(E, T)* is the Fermi-Dirac distribution (at temperature *T*). Throughout the analysis *T*_b_ is also assumed to be the temperature of the superconducting electrodes. We use the differential conductance *G* to represent the transport properties, which is given by


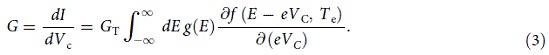


The sub-gap leakage is empirically described by the Dynes parameter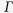, which is introduced in the Dynes model^20^. In the Dynes model the superconducting DOS is described by


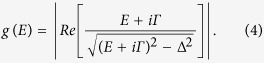


**Figure 1:**
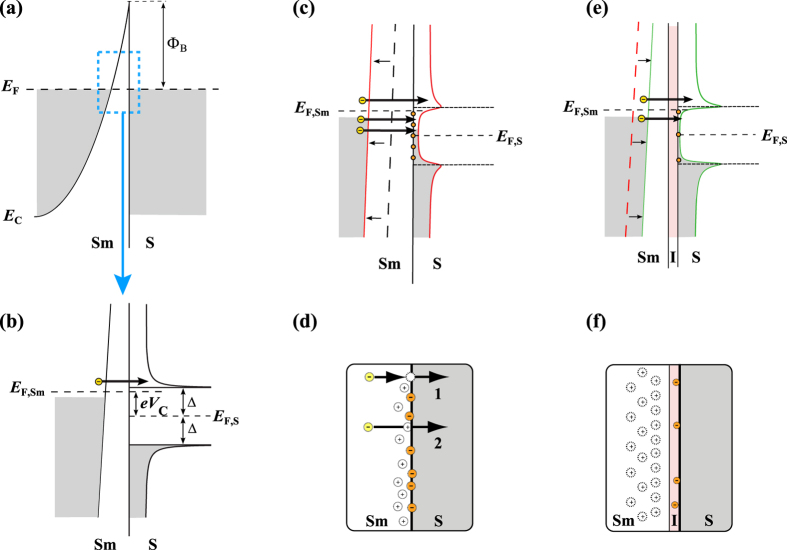
Energy bands and electronic transport at the interface. (**a**) Energy band diagram showing an ideal Sm-S Schottky barrier, where the barrier height is defined as Φ_B_ = E_c_ −E_F_, the difference between the semiconductor conduction band edge E_c_ and Fermi level E_F_ at the interface. (**b**) A blow-up of (**a**) around the Fermi level revealing the ideal superconductor density of states (DOS) and superconducting gap Δ. Hot electron tunnelling from semiconductor to the superconductor is also illustrated. (**c**) Non-ideal Sm-S junction with negatively charged surface states at the interface (orange filled circles), which cause a shift of the conduction band and broaden the effective superconductor DOS by introducing sub-gap leakage channels due to unoccupied trap states close to the Fermi level. (**d**) Real space illustration of the leakage channels. Channel 1 illustrates the leakage through the interface trap states [the same as in (c)] and channel 2 the donor induced leakage channels. (**e**) Energy band diagram and (**f**) real-space illustration of the passivation effect of the insulating layer I. Leakage paths are diminished and Schottky barrier thickness is reduced. The latter being equivalent to Fermi-level depinning.

By setting *Γ* = 0 we obtain the ideal sharp BCS (Bardeen-Cooper-Schrieffer) DOS without any states in the superconducting gap (see Fig. 1b). Non-zero *Γ* smears the DOS around the gap edges and produces finite amount of states in the superconducting gap Δ (see Fig. 1c).

We determine the device parameters *G*_T_ and Δ from the electrical measurements described in the Supplementary Material. We evaluate the quality of the tunnel junction interfaces using the ratio *G*_0_/*G*_T_, where *G*_0_ is the zero bias conductance of the junction. *G*_0_ is temperature dependent and should be investigated at sufficiently low-temperature, well below the superconductor critical temperature so that the sub-gap current is not dominated by thermally excited quasiparticles. Note that at *k*_*B*_*T* ≪ Δ the *G*_0_/*G*_T_ -ratio is numerically equivalent to the Dynes parameter *Γ/*Δ. In this work, we refer to the low temperature saturated *G*_0_/*G*_T_ as the junction leakage.

Figure 3 main shows *G*_0_*/G*_T_ as a function of temperature. The oxide junction sample Si-2 shows clear reduction in the sub-gap conductance in comparison to the un-oxidized control sample Si-1. For the strained epitaxial sample we can observe a similar trend: the un-oxidized strained sample originally reported in ref. 19 has significantly larger sub-gap leakage than the oxidized strained sample sSi. Conductance as a function of junction bias is shown in Fig. 3 inset. In addition of the data from samples Si-1 and Si-2 the inset also shows data measured from the Ar plasma treated sample. The small local maxima in the conductance at zero voltage bias for the oxidized sample Si-2 is a common indication of Andreev tunnelling^21^,^22^,^23^, also seen in high transparency high quality NIS tunnel junctions^24^.

### Cooling performance

**Figure 2:**
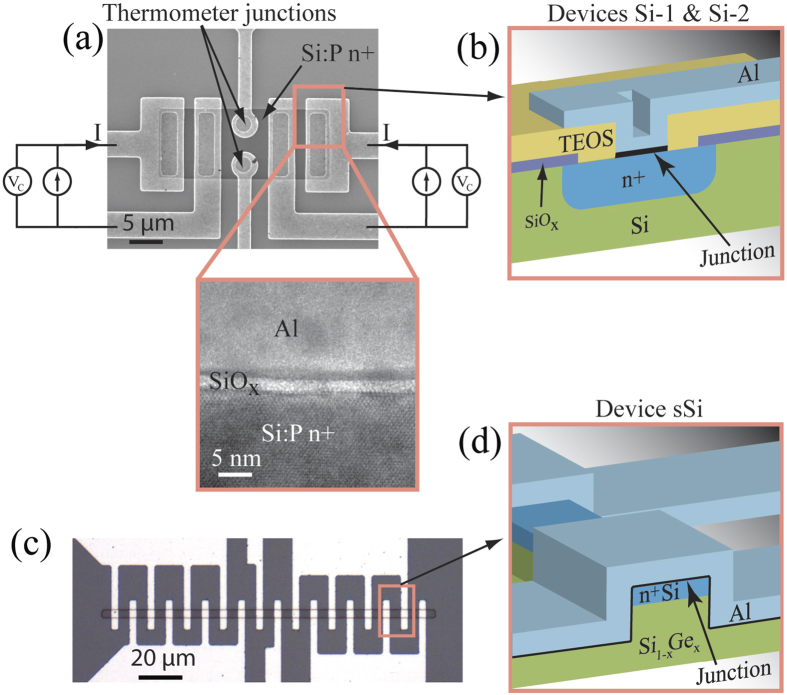
Cooler devices. (**a**) SEM and TEM micrographs of unstrained doped well samples. The Si:P island is visible as a darker shade under the junctions and electrodes in the SEM picture. The high resolution TEM micrograph shows the SiO_x_ layer at the junction interface for sample Si-2. (**b**) Schematic cross-section of unstrained doped well samples Si-1 and Si-2. (**c**) Optical microscope image of the strained epi-layer sample sSi and (**d**) schematic cross-section. For all devices the semiconductor (Sm) is doped silicon and the superconductor (S) is aluminium.

The cooling power of superconducting tunnel junction degrades as a function of the sub-gap leakage. This degradation arises from the low energy tunnelling within the gap, which, as stated above, is empirically described using the Dynes density of states. Operated as a cooler, the degenerately doped Si island was cooled through two pairs of symmetric SmIS (Si-SiO_x_-Al) junctions or Sm-S junctions biased in parallel and the island temperature *T*_*e*_ was measured with an independent pair of high resistance junctions used as a thermometer as illustrated in Fig. 2a. The thermometer was calibrated at zero cooler bias, where *P*_c_ = 0 and *T*_e_ *=* *T*_b_ and was then used to determine *T*_e_ at non-zero bias resulting in Fig. 4a,c,d for samples Si-1, Si-2 and sSi, respectively. The measurement setup and thermometry calibration is described in the Supplementary Material.

The cooling power of a tunnel junction can be expressed as^10^


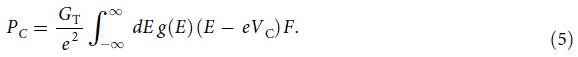


The cooling power *P*_c_ opposes inherent thermal coupling and heating mechanisms which need to be included in a heat balance equation to determine the resultant electron temperature. To model our experimental results we have adopted the thermal model utilized in ref. 19, and the results are shown as dashed and full lines in Fig. 4a–f. A full model description together with the fit parameters can also be found from the Supplementary Material.

The electron temperatures obtained from the thermometer and model are in good agreement for all devices, other than minor deviations for the measurements taken at low *T*_b_. These deviations we attribute to leakage currents in the thermometer junctions, not included in the thermal model. We also compare the conductance curves with those of the model, as shown in Fig. 4b,d,f, which show that our model compares well to the experimental conductance characteristics of the coolers.

For the sSi cooler we get an impressive cooling down to *T*_e_ = 90 mK from *T*_b_ = 300 mK. The total power load for the sSi cooler is lower when compared to our unstrained Si devices due to factor of ~40 lower electron-phonon coupling^15^,^19^ and reduced Joule heating. Compared to previous results on sSi devices with the same geometry and Joule heating^19^, our new oxide junction device shows a large improvement in cooling performance attributed to both the reduced sub-gap leakage and the comparably higher *G*_T_ (see Table 1).

To determine how our tunnel junction cooling performance compares to that of an ideal tunnel junction, we have studied the ratio *P*_c_ (*Γ* )/P_max_ which can be considered as a cooler junction figure of merit. *P*_max_ is the maximum cooling power obtained at optimal *V*_*C*_ and *Γ* *=* *0*, i.e, *P*_max_ is linked to the ideal BCS DOS. Optimal bias and *P*_*max*_ have closed form from analytical approximations, *V*_C_ *~* 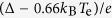 and *P*_max_ ~ 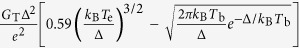, but here these parameters were determined numerically.

In Fig. 5, we have plotted *P*_c_(Γ*)/P*_max_ as a function of *Γ/*Δ and temperature *T*_e_ (with *T*_e_ = *T*_b_). This plot illustrates that, for a given *Γ/*Δ, there is a minimum temperature where one can expect cooling, which serves as an indication of the tunnel junction quality required for cooling applications. As the electron temperature is reduced, for a given junction leakage *Γ/*Δ, the cooling drops to zero (black line) at a temperature given by the relation *T*_e_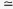 *T*_c_ 2.5 (*Γ/*Δ)^2/3^ 25. For poor quality junctions (high *Γ/*Δ) the zero figure of merit is reached at higher temperatures. Therefore, to reach low electron temperatures, the figure of merit must remain high at low temperatures and this can only be achieved with high quality junctions (low *Γ/*Δ). The cooler junction figure of merit drops with temperature because the useful cooling power depends on the spread of the Fermi distribution, which reduces with lowering the temperature, whereas the sub-gap leakage provides a heating component to the power integral in equation (5), which is nearly constant with temperature. Thus, leakage quickly becomes dominant at low temperature unless *Γ* is low.

## Discussion

**Figure 3:**
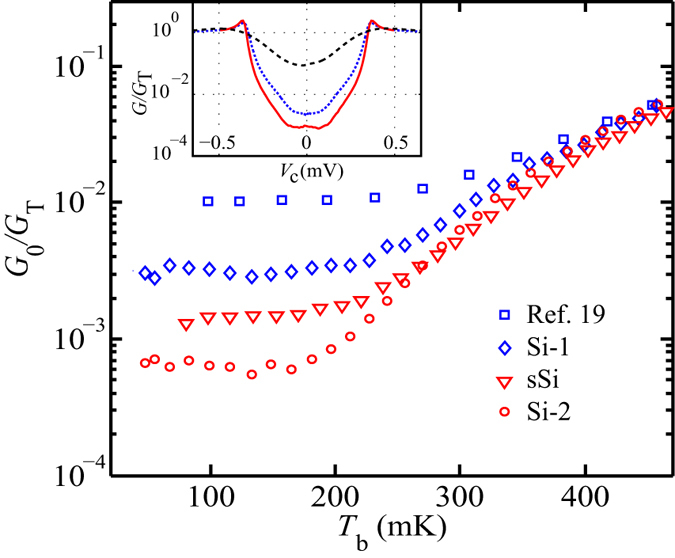
Differential conductance. Temperature dependence of the normalized differential zero-bias conductance G_0_/G_T_ measured from different samples. Inset: Normalized conductance curves G/G_T_ as a function of voltage bias measured from samples Si-1 (blue dotted line) and Si-2 (red line) at T_b_ = 30 mK. The black dashed curve shows the conductance curve of the Ar plasma degraded junction.

**Figure 4:**
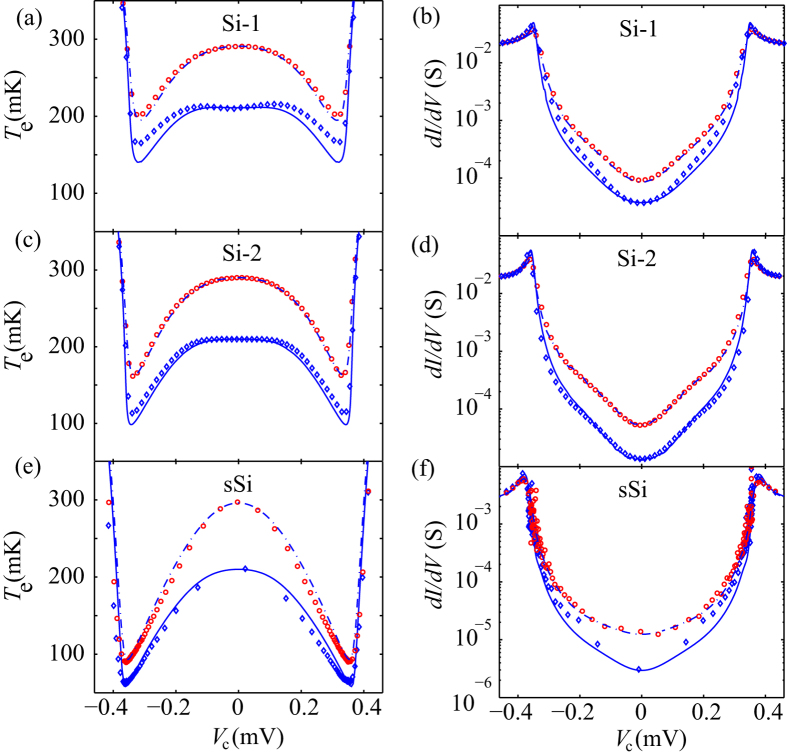
Cooling and electronic properties. (**a,c,e**) Electron temperature and (**b,d,f**) differential conductance of different samples as function of junction bias voltage. Symbols are experimental data and the solid and dashed curves are fits to the model. Electron temperature equals to the bath temperature at V_C_ = 0.

The similar *R*_c_ for samples Si-1 and Si-2, with 1.1 kΩμm^2^ and 1.35 kΩμm^2^ respectively, seems at a first glance a counter intuitive result, since in addition to the Schottky barrier sample Si-2 also has the oxide barrier, which should significantly increase the tunnel resistance between Si and Al in comparison to sample Si-1. Firstly, we suggest that the oxide layer passivates the surface causing reduction in the interface state density and, thereby, reduction of the negative charge at the interface, which leads to lowering (and thinning) of the Schottky barrier. This is equivalent to the Fermi-level de-pinning effect that such thin insulator layers have been shown to enable^26^. Note that sample Si-1 has higher sub-gap leakage than sample Si-2 (Fig. 3) and, therefore, we postulate that the opening of the superconducting gap creates a sensitive instrument to probe the interface states giving access to information that is hidden when investigating the contact resistance between semiconductor and metal only in the normal non-superconducting state. Similar interfacial effects have been also investigated in fully metallic junctions^27^,^28^. In addition to the interface state effect the oxide can create a high density donor layer in Si just next to the oxide (see Fig. 1f). This occurs because the low solubility of phosphorous in SiO_x_ causes repelling of the dopants into the Si during the oxidation step. The effect is known as the dopant segregation effect^29^ and it has been utilized in many nano-device fabrication recipes^30^,^31^. As the oxidation temperature (550 ^o^C) is too low for strong phosphorous diffusion the high density donor region forms and this further reduces the Schottky barrier. The combination of the Fermi-level de-pinning and the segregation effects virtually offset the impact of the oxide barrier, leading to only a small increase in the tunnel resistance - from 1.1 kΩμm^2^ (sample Si-1) to 1.35 kΩμm^2^ (sample Si-2). Without these effects, the oxidation would create very high tunnel resistance and the junction would be relatively useless as a cooler or bolometer element. Because of this expectation, it has taken 14 years from the first studies on the Sm-S cooler^6^ to realize a truly effective cooling performance in a silicon-based tunnelling junction.

Note that the behaviour of the Ar plasma-treated sample fully supports the above interpretation with regard to the interface traps and the superconductor being a useful probe of such states. The Ar treatment damages the semiconductor surface and increases the number of the traps, so for this sample *R*_c_ and sub-gap leakage should be high. Indeed, this is precisely what we observe: we get a very large *R*_c_ of 10 kΩμm^2^ and sub-gap leakage is almost one order of magnitude higher compared to the process without Ar plasma (see the inset of Fig. 3).

The oxide barrier also confers other enhancements on the tunnelling cooling process. Our experimental results are well explained using the Dynes DOS, and the reduced sub-gap leakage clearly improves the junction performance. We infer that low-energy sub-gap tunnelling processes in Sm-S junctions occur through the interface states and dopant induced channels (Fig. 1c,d). Their proximity to the superconductor may create gap states in the superconductor or local high transparency channels, which results in increased single particle or 2-particle tunnelling (Andreev reflection)^21^. Adding an oxide barrier effectively reduces the number of these local channels, both through the reduction of interface states and the retraction of dopants at the interface, Fig. 1f. Compared to previous works^6^,^16^,^17^,^19^ (see also Table 1), our findings clearly show that with our interface engineering we have significantly reduced both the high contact/tunnel resistance and high sub-gap leakage of Sm-S tunnel junctions – results that guarantee high cooling power.

**Figure 5:**
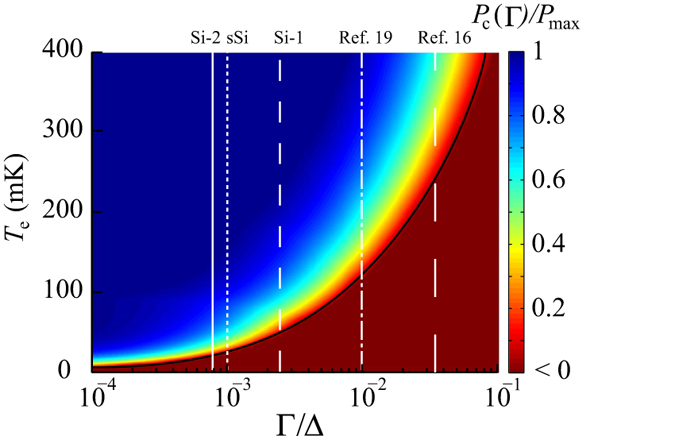
Figure of merit of junction coolers. Contour plot of figure of merit P_c_(Γ )/P_max_ as a function of Dynes parameter Γ/Δ and electron temperature T_e_. P_c_(Γ )/P_max_ < 0 corresponds to heating. For Γ/Δ > 6 × 10^-3^ there is no cooling power at T_b_ < 100 mK, which also is observed for the earlier work listed in Table 1. The white vertical lines indicate the Γ/Δ for Sample Si-1, Sample Si-2, Sample sSi, ref. 19 and 16.

A closer study of the results in Fig. 5 reveals that the cooling power, *P*_c_, for the oxide junction sample Si-2 is ~ 90% of the ideal cooling power at *T*_b_ ~ 100 mK, based on the value of *Γ/*Δ = 8 × 10^−4^ that we extracted from the transport measurements. The Sm-S device Si-1 is further from the ideal maximum performance with ~55% of the ideal cooling power at the same temperature (with *Γ/*Δ = 2.5 × 10^−3^). Both of these results are significant improvements compared to previous work on Sm-S coolers^6^,^16^,^17^,^19^, where no cooling power was available at these temperatures (for *Γ/*Δ ≥ 10^−2^, see also Table 1). For the sSi sample, the oxide junction quality showed a sub-gap leakage of *Γ/*Δ = 1 × 10^−3^, which is an order of magnitude better than obtained from previous sSi devices^19^, indicated in Table 1 and Fig. 5. From the comparison in Fig. 5, this device would be expected to have a useful cooling power *P*_c_ down to *T*_b_ ~ 35 mK, however here the load from electron-phonon (e-ph) coupling and Joule heating in this particular device limits the observed cooling.

The origin of the larger sub-gap leakage and significantly higher characteristic resistance *R*_c_ in the sSi device in comparison to the devices Si-1 and Si-2 is not known. We speculate that dislocations and mesa type structure can introduce more leakage paths. The dopant density is slightly lower in device sSi (2.7 × 10^19^ cm^−3^) than in devices Si-1 and Si-2 (4 × 10^19^ cm^−3^), but this alone cannot explain the order of magnitude difference in *R*_c_. On the other hand, once again we can observe correlation between the magnitude of *R*_c_ and sub-gap leakage. If we can obtain similar junctions for sSi as in device Si-1, on the basis of our simulations, we would observe cooling from 300 mK to 46 mK.

It should be noted that another potential contribution to the sub-gap leakage comes from coupling to environmental noise^32^, which can also be empirically described by the Dynes model. Note also that doped semiconductors have significantly higher resistance than metals do and, therefore, noise coupling can be more serious issue in Sm-S devices than in NIS devices. This can be observed as a direct heating which can lead to a saturation of the low-temperature sub-gap conductance, as observed in Fig. 3. Therefore, the Γ-values found in this work must be considered as the upper limit values.

Large resistivity and low e-ph coupling of doped Si also provides the possibility for extremely high sensitivity THz detection. This is especially true for strained Si where ultra-low e-ph coupling values can be achieved^15^ and very recently the first strained Si-based S-Sm-S THz bolometer has been demonstrated^33^. The junction technology introduced here can be directly adapted to such device to create a bolometer with improved characteristics.

Si-based cooler technology has an advantage of allowing a wider selection of superconductor materials, since in contrast to metal-based NIS coolers the quality of the junction interface is mainly determined by the cleaning/oxidation process of the normal (semiconductor) island. It is therefore plausible that larger gap superconductors (e.g. Nb, V) could be employed to initiate cooling in doped Si from temperatures as high as 1.5 K. Critically, the lower electron-phonon coupling in silicon than in metals, with further very large reductions possible with increased strain^14^,^15^, could make silicon-based cooling technology superior to its all-metal counterparts.

In summary, we have demonstrated high transparency and low sub-gap leakage Sm-S cooler junctions by engineering the Sm-S interface by an insulator tunnel barrier between the S and Sm electrodes. Despite the introduction of the insulator barrier layer to the Schottky junction, the resistance of the superconductor-insulator-semiconductor cooler junction remains low. This unanticipated result was linked to the Fermi level de-pinning and dopant segregation effects that strongly affect the junction properties at the nanoscale. The insulator barrier layer significantly reduced the sub-gap leakage channels associated with interface states and dopants in non-oxidized Schottky barrier devices. By investigating a damaged surface sample we showed that the interface states at the Sm-S interface give a large contribution to the sub-gap leakage current. Therefore, a superconducting electrode can act as a sensitive probe to the metal-semiconductor surface states. Due to high transparency and low leakage the present junctions show unparalleled cooling power performance, in comparison to previous works on Sm-S coolers. Indeed, the demonstrated performance is comparable to that of high power NIS coolers^34^, and the prospects posed by the first Sm-S cooler investigations 14 years ago^6^ are accessible in practical devices. By adapting the new junctions in strain-engineered silicon coolers, where electron-phonon coupling is strongly reduced by strain, we also demonstrated efficient electron temperature reduction from 300 mK to below 100 mK by the electronic cooling process. In this work we utilized Al as the superconductor, which limits the operation to sub-1 K temperatures. The demonstrated Si-based cooler technology can enable cooling from above 1 K by the utilization of higher gap superconductors, with transition temperatures above 1 K.

## Methods

The samples in this study were fabricated on Si substrates utilizing UV lithography, dopant implantation, wet and plasma etching, epitaxial growth and different film deposition techniques. The details of the processes are described in the sample fabrication section of the Supplementary Material.

The transport measurements at low temperature have been performed in a dilution refrigerator down to a temperature of *T*_b_ = 30 mK. The refrigerator was equipped with measurement lines containing a combination of shunting and dissipative filters.

The *I-V* characteristics of both cooler and thermometer junctions were measured with a standard four-point measurement scheme. The tunnel junction resistance *R*_T_ of our devices is given by the asymptotic resistance at voltages *V* ≫ *Δ/e* and the superconducting gap *Δ* is defined by the threshold voltage Δ*/e* from the *I-V* characteristics at *T* ≪ *T*_c_. The conductance curves were obtained through differentiating the measured *I-V* data.

Hall bar measurements at 10 K provide values for the mobility, carrier density and sheet resistance of the sample under test. The carrier density *N* is calculated using the measured sheet density and an estimated implant layer thickness of 150 nm. From the sheet resistance we can obtain the Si island resistance *R*_sm_ of the samples in this work.

## Additional Information

**How to cite this article**: Gunnarsson, D. *et al.* Interfacial Engineering of Semiconductor–Superconductor Junctions for High Performance Micro-Coolers. *Sci. Rep.* **5**, 17398; doi: 10.1038/srep17398 (2015).

## Supplementary Material

Supplementary Information

## Figures and Tables

**Table 1:** Sample parameters and comparison with previous works on Sm-S junctions.

Ref.	*R*_c_ (kΩμm^2^)	*Γ/*Δ	*P*_c_ @ 0.3 K (pW/μm^2^)	*P*_c_@ 0.1 K (pW/μm^2^)
6	36	—	0.03	Heat
16	2000	3.5 × 10^−2^	~0	Heat
19	100	1.0 × 10^−2^	0.02	Heat
19	10	1.5 × 10^−2^	0.14	Heat
sSi	31	1.0 × 10^−3^	0.04	0.006
Si-1	1.1	2.5 × 10^−3^	1	0.13
Si-2	1.35	8 × 10^−4^	0.86	0.14

See Supplementary Table 1 for all parameters of devices Si-1, Si-2, and sSi. The Ref. 19 strained sample in Fig. 3 is the one with Rc = 100 **k**Ωμm^2^. Heat means that the junctions introduce heating instead of cooling.
