# Supplementary material for: Interfacial Engineering of Semiconductor–Superconductor Junctions for High Performance Micro-Coolers
Source: Sci Rep. 2015 Dec 1;5:17398. doi: 10.1038/srep17398 (PMC4665018; doi:10.1038/srep17398)
Supplement: Supplementary Information [file srep17398-s1.pdf]

Supplementary Information

Interfacial Engineering of Semiconductor–Superconductor Junctions for High Performance Micro-Coolers

D. Gunnarsson[1], J.S. Richardson-Bullock[2], M.J. Prest[2], H. Q. Nguyen[3], A.V. Timofeev[1], V. A. Shah[2], T.E. Whall[2], E.H.C. Parker[2], D.R. Leadley[2], M. Myronov[2] and M. Prunnila[1,*]

[1]*VTT Technical Research Centre of Finland, P.O. Box 1000, FI-02044 VTT Espoo, Finland*

[2]*Department of Physics, University of Warwick, Coventry CV4 7AL, UK*

[3]*Low Temperature Laboratory (OVLL), Aalto University School of Science, PO Box 13500, FI-00076 Aalto, Finland*

This document is present in conjunction with the manuscript entitled "**Interfacial Engineering of Semiconductor– Superconductor Junctions for High Performance Micro-Coolers**" It provides further details on the sample fabrication, experimental methods and data analysis in support of the conclusions drawn in the parent manuscript. (Total page count 7)

*mika.prunnila@vtt.fi

1. Device fabrication

Doped well devices: Experimental sample preparation began with a standard low resistivity (1-10 Ω cm p-type) (100) 6" silicon wafer which underwent a blanket SiO_2 deposition, followed by photolithography to define regions for dopant implantation. Windows in the surface oxide were opened using a buffered HF solution before phosphorus ion implantation and activation anneal to give a surface doping concentration of N_D=4×10^{19} cm^{-3}. A second round of oxide deposition, photolithography and chemical etch was carried out in order to define the junction areas. After 2 % HF dip (30 s) the wafers were quickly rinsed and dried and loaded to the sputtering chamber. A thin oxide tunnel barrier was grown via an in-situ oxidation at 300 Torr and 550 °C for 10 minutes for Si-2 samples. For the Ar plasma treated sample we used 15 s Ar treatment at 900 eV. Aluminium was then deposited, patterned and etched to form the superconducting electrode and contact metal. The cooler geometry consisted of an electronically active region with dimensions 10 μm by 25 μm, a pair of circular central thermometer junctions, each with a diameter of 2 μm, with two 9 μm by 2 μm cooler junctions either side, giving a combined cooling junction area of 72 μm^2.

Strained epi-layer devices: For the strained silicon sample, biaxial tensile strain was induced via lattice mismatch between silicon and the silicon-germanium alloy. As with the doped well devices, we used a standard low resistivity (1-10 Ω cm p-type) (100) silicon wafer. Firstly, a linearly graded $Si_{1-x}Ge_x$ layer was grown with the germanium content x increasing from 0 to 0.22 over 2 μm. This layer was relaxed to the lattice spacing of the silicon germanium alloy and the upper interface was relatively free from crystal dislocations as these were confined within the lower section of the layer. A constant composition layer of 500 nm $Si_{0.78}Ge_{0.22}$ was deposited to ensure good material quality. Finally, a 30 nm silicon layer was grown, conforming to the lattice spacing of the alloy below. This layer was epitaxially doped to N_D=4×10^{19} cm^{-3}. The silicon was found to be under approximately 0.95% of tensile strain as confirmed by X-ray diffraction analysis. The mesa was patterned by photolithography followed by plasma etching. In the case of the cooler structures this created a raised rectangular pillar approximately 100 nm tall with lateral dimensions 148 μm by 4 μm. The active layer was confined within the top 30 nm, thus the degenerately doped silicon was effectively isolated and its geometry was well defined. The native oxide was removed using 1% HF before the thin oxide tunnel barrier was grown via an in-situ oxidation at 200 Torr and 550 °C for 10 minutes. Aluminium was then deposited, patterned and etched to form the superconducting electrodes and contact metal. For the cooler structure, the geometry consisted of an electronically active region with dimensions 148μm by 4 μm, the central thermometer was formed of two 4 μm by 4 μm junctions, with eight 6 μm by 4 μm cooler junctions either side, giving a combined cooling junction area of 384 μm^2.

Both the doped well and strained device designs adopted an array of interdigitated parallel junctions. This geometry both improves the quasiparticle thermalisation and reduces the series resistance of the silicon R_{sm} which reduces Joule heating.

Table 1: Device parameters. Dopant concentrations were confirmed by Hall measurements and R_C was extracted from junction I-V measurements. Volume, Ω and electron-phonon coupling factor, Σ were not required when fitting the data for the Si-1 and Si-2 samples as described in section 4.

Sample	Si-1	Si-2	s-Si
**Steps prior Al deposition**	HF-dip	HF-dip<br>oxidation	HF-dip<br>oxidation
**Sample Parameters**			
N_D **(x10^{19} cm^{-3})**	4	4	2.7
R_c **(kΩµm^2) @ 300 K**	1.1	1.35	31
G_0/G_T **@ 30 mK**	0.0025	0.0008	0.001
**Fit Parameters**			
R_T **(Ω)**	67	76	440
R_{sm} **(Ω)**	29	29	81
**Δ (µeV)**	194	194	190
**Γ/Δ**	0.0025	0.0008	0.001
**B**	0.11	0.08	0.03
**Ω (m-^{3})**			1.78x10^{-17}
**Σ (W m^{-3} K^{-6})**			3x10^7

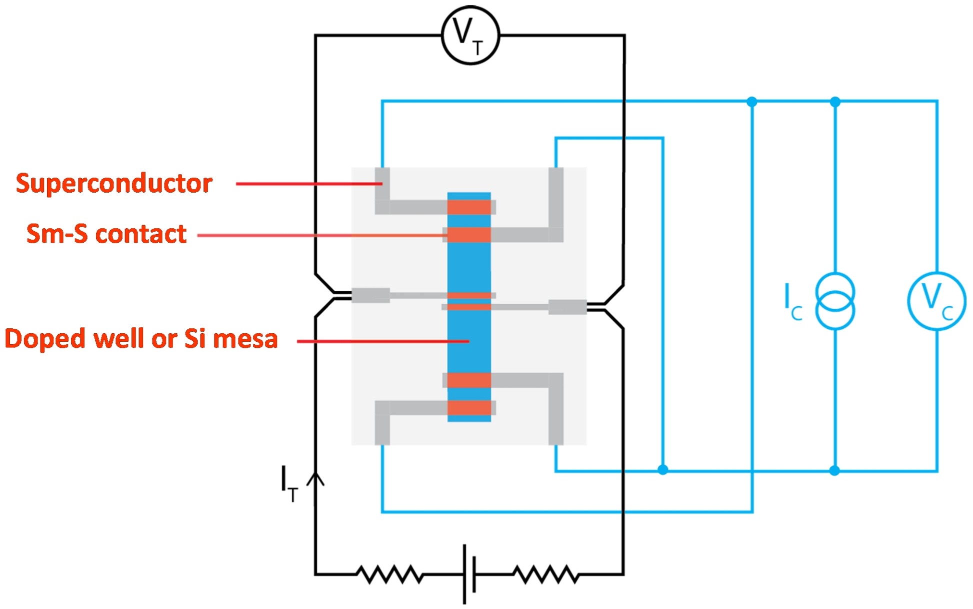

Figure 1: Illustration of electronic measurement scheme of the cooler devices. I_T (V_T) is the thermometer current (voltage) and I_C (V_C) is the cooler current (voltage). Similar approach was used for the electron-phonon coupling measurement.

2. Electron Cooling measurements

Whilst the geometry and design of the electron coolers varied between devices, a generic layout is presented in figure 1 to illustrate the experimental setup. The cooler devices presented in this work utilised multiple junction coolers and a single, smaller double junction positioned symmetrically between the two coolers for electron temperature thermometry.

The cooler Sm-S junction pairs were connected in parallel and a range of biases were applied. Figure 1 illustrates current bias, but in the measurement both current and voltage were measured simultaneously and the nature of the bias (voltage/current) depended on the bias point due to the strong nonlinearity of the Sm-S junction. Simultaneously, the thermometer junctions were biased at optimum sensitivity using a high impedance battery powered current source and the voltage drop V_T was amplified using a floating differential voltage amplifier. The cooler voltage sweeps were repeated for a set of bath temperatures T_b and the thermometer junction voltage was calibrated against a RuO thermometer at $V_C = 0$, where the electron temperature is equal to that of the surrounding bath T_b. This is the standard procedure for junction thermometry [1]**.** See the calibration section of this document for further details.

3. Modelling

The cooling power of a junction is balanced against a number of heating mechanisms, principally the heat flow between the lattice phonons and the electron gas, mediated by the electron-phonon coupling. This is given by the equation

$$P_{\mathrm{e-ph}} = \Sigma\Omega\left(T_{\mathrm{e}}^{6} - T_{\mathrm{b}}^{6}\right) \quad (1)$$

with Ω being the cooled volume and Σ the material specific coupling constant, which has been studied in previous work [2]. Additional heating power

$$P_{\mathrm{J}} = -I^{2}R_{\mathrm{Sm}} \quad (2)$$

is included to account for the Joule heating as a result of the semiconductor resistance R_{Sm}, along with a term

$$P_{\mathrm{QP}} = -\,\beta\,(IV + P_{\mathrm{c}}) \quad (3)$$

describing the fraction of power β returned to the superconducting electrode via quasiparticle effects such as back-tunnelling and recombination in close proximity to the junction. [3, 4].

The equilibrium state of these power flows is described by the heat balance equation

$$P_{\mathrm{c}}(V_{\mathrm{c}},T_{\mathrm{e}}) + P_{\mathrm{e-ph}}(T_{\mathrm{e}}) + P_{\mathrm{J}}(V_{\mathrm{c}},T_{\mathrm{e}}) + P_{\mathrm{QP}}(V_{\mathrm{c}},T_{\mathrm{e}}) = 0 \quad (4)$$

which can be solved for electron temperature for a given cooler voltage. Here P_c is the junction cooling as referred to in the main paper.

4. Electron-phonon coupling

The electron-phonon coupling heat flow $P_{\mathrm{e-ph}}$ is typically calculated using the known volume Ω and the coupling constant of the cooled material Σ [5]. In this work, this procedure is followed for the strained samples. For the doped well samples the exact thickness is difficult to determine accurately as the doping profile arises from the implantation and annealing/activation step. Therefore, we determined $P_{\mathrm{e-ph}}$ from electron heating measurement described below.

The electron-phonon coupling test device consisted of a long large area bar (200 μm x 5 μm) which was fabricated on the same wafer with the cooler devices. In the experiments a pair of thermometer junctions half way along the bar were used to record the electron temperature T_e as a function of the applied Joule heating power $P = P_{\mathrm{e-ph}}$ produced by electric current (this is the well-known hot-electron

effect observed at low temperatures [1,2,6]). The electron thermometry was similar as in the electron refrigeration measurements for the cooler devices (see section 5 and also Figure 1). To generate a virtually continuous data set for P_{e-ph}, which can be used at arbitrary bath temperature T_b and T_e, we measured electron temperature response to the Joule heating at several T_b and then generated the electron-phonon thermal conductance

$$G_{e-ph} = \frac{dP}{dT_e} \tag{5}$$

from the data in the spirit of Ref. [2]. The result is shown in figure 2. We can observe that G_{e-ph} follows closely to T^5 power law as expected for a disordered many-valley semiconductor [2]. To find P_{e-ph}, when solving equation (4), we simply integrated the measured G_{e-ph} between the limits of T_e and T_b to extract P_{e-ph} as a function of electron temperature. This result was then scaled for the area of a specific cooler and used in (4), allowing us to accurately model the balance of heat mechanisms in the device. In this way, integration of (5) was used to experimentally determine P_{e-ph}.

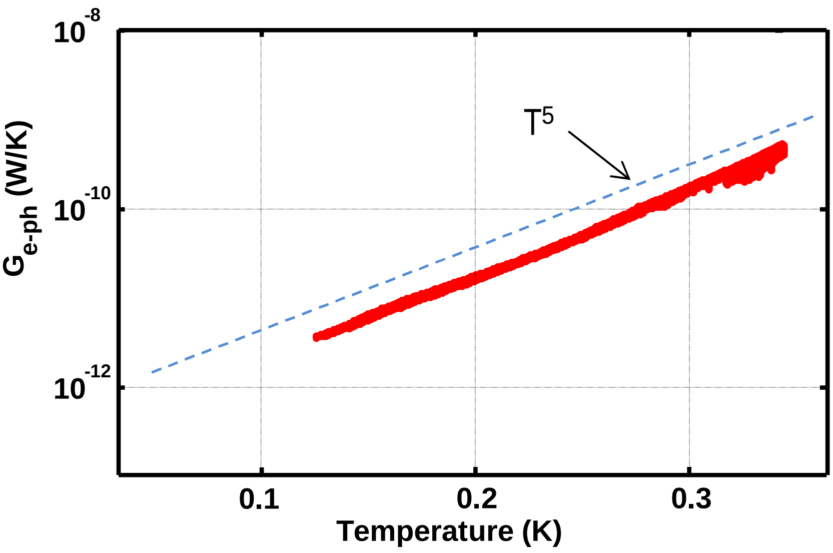

Figure 2: Electron-phonon thermal conductance as a function of electron temperature (red dots). T^5 dependency is illustrated by the dashed line.

5. Thermometer Calibration

We biased the small area thermometer tunnel junctions with a small constant current (0.1-1 nA). The measured thermometer voltage V_T at $V_C = 0$, where the electron temperature is equal to that of the surrounding bath T_b, produced a calibration curve such as that shown in the left panel of figure 3.

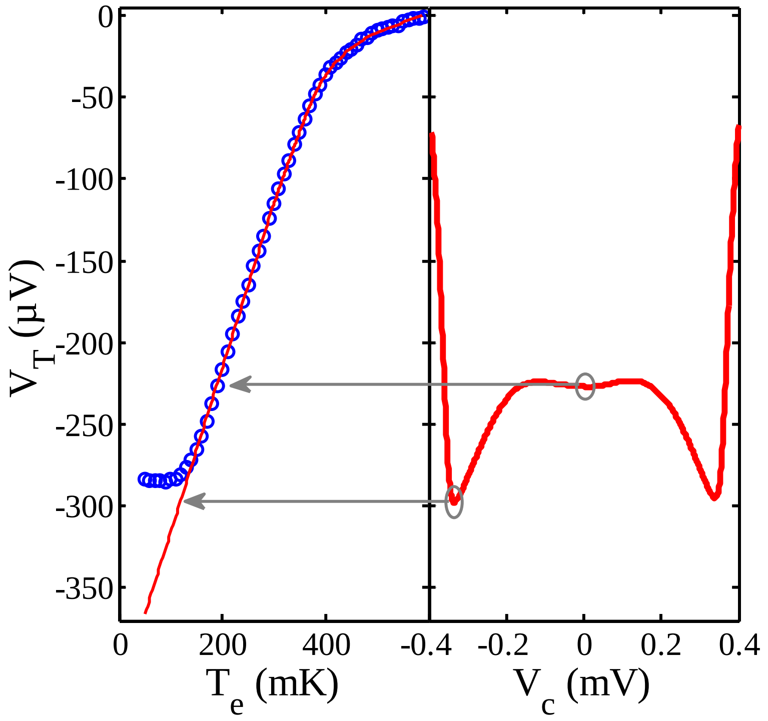

Figure 3: (Left) Thermometer calibration curve example for doped well cooler device. Blue circles are thermometer voltage points V_T measured for T_e=T_b over a range of bath temperatures T_b. Red curve is the calibration curve that maps V_T to T_e. (Right) Thermometer voltage as a function cooler voltage at T_b = 190 mK.

The junction thermometers typically become less sensitive at lower temperatures due to sub-gap leakage, power dissipation from the RF noise of the environment and heating from the thermometer bias current. These effects lead to saturation of the thermometer and the voltage no longer drops with bath temperature. However, during the electron refrigeration by the large cooler junctions, the parasitic heating effects are offset by the cooling power, so that the voltage response of the thermometer continues towards lower temperatures (see the right panel of figure 3). For this reason we use linear extrapolation to lower temperatures and lower thermometer probe voltages.

We also investigated an alternative calibration method called the isotherm method, which has been used successfully by other groups [7, 8] and relies on comparing the measured data to calculated I-V curves using parameters extracted from a fit to a higher temperature measurement. The measured current-voltage data was superimposed on a series of theoretical isotherm curves generated from equation (1), using the parameters of the device under test. Each point of intersection between the experimental data and an isotherm yields the electron gas temperature in the semiconductor island at that particular cooler junction voltage. Figure 4 compares the results for the strained silicon device as determined using the current biased junction thermometry method and the isotherm method. The isotherm method is not very accurate at low biases, so we have not included low voltage results for the isotherm method. The agreement of the two methods is very good (see figure 4) and in the main paper we resort to the junction thermometry method.

.

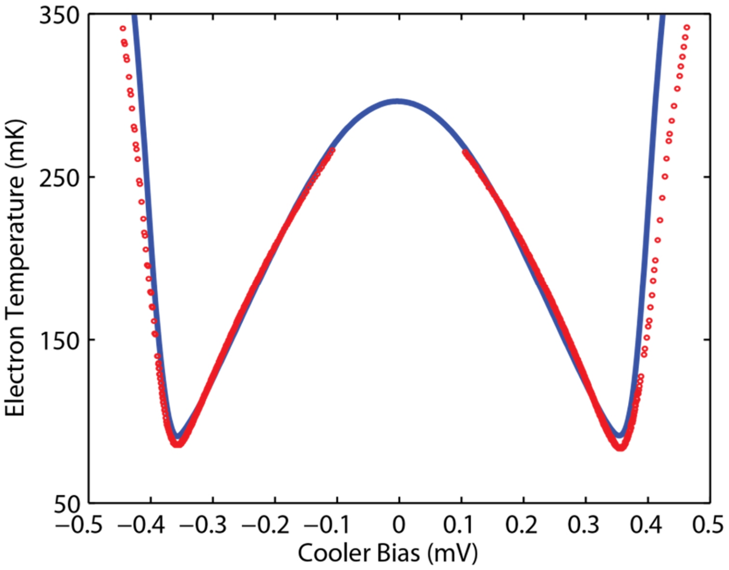

Figure 4: Extracted electron temperature T_e versus applied cooler bias voltage V_c, as extracted via junction thermometry (solid blue line) and isotherm method (red circles).

References

[1] Nahum, M. and Martinis, J. M. Ultrasensitive-hot-electron microbolometer. *App. Phys. Lett.* **63**, 3075 (1993).

[2] Muhonen, J. T., J. T. Prunnila, M. Gunnarsson, D. Shah, V. A. Morris, R. J. H. Dobbie, A. Myronov, M. T. E. Whall, Parker, E. H. C. and Leadley, D. R. Strain dependence of electron-phonon energy loss rate in many-valley semiconductors. *Appl. Phys. Lett.*, **98**, 182103 (2011).

[3] Fisher, P. A. Ullom, J. N. and Nahum, M. High-power on-chip microrefrigerator based on a normal-metal/insulator/superconductor tunnel junction. *Appl. Phys. Lett.*, **74**, 2705 (1999).

[4] Ullom, J. N. Quasiparticle behavior in tunnel junction refrigerators. *Phys. B Condens. Matter* **284–288**, 2036–2038 (2000).

[5] Prest, M. J. Muhonen, J. T. Prunnila, M. Gunnarsson, D. Shah, V. A. Morris, R. J. H. Dobbie, A. Myronov, M. T. E. Whall, Parker, E. H. C. and Leadley, D. R. Strain enhanced electron cooling in a degenerately doped semiconductor. *Appl. Phys. Lett.*, **99**, 251908 (2011).

[6] Wellstood, F. C., Urbina C. and Clarke J. Hot electron effects in metals. *Phys. Rev. B.* 49 5942 (1994)

[7] Rajauria, S. Luo, P. S. Fournier, T. Hekking, F.W. J. Courtois, H. and Pannetier, B. Electron and Phonon Cooling in a Superconductor-Normal-Metal-Superconductor Tunnel Junction. *Phys Rev. Lett*. 99 047004 (2007)

[8] O'Neil, G. C. Lowell, P. J. Underwood, J.M. and Ullom, J. N. Measurement and modeling of a large-area normal-metal/insulator/superconductor refrigerator with improved cooling. *Phys. Rev. B* 85 134504 (2012)
